# Hemodynamic Consequences and Clinical Outcomes With Intravenous Lidocaine Infusion in Patients With Atrial Fibrillation

**DOI:** 10.1111/jce.70321

**Published:** 2026-03-19

**Authors:** Andrea N. Keithler, Zameer Abedin, Deborah Furman, Luke Schwarz, Marc C. Engels, Roger A. Freedman, Klitos Konstantinidis, H. Immo Lehmann, Ravi Ranjan, Benjamin A. Steinberg, T. Jared Bunch

**Affiliations:** ^1^ Department of Clinical Cardiac Electrophysiology Division of Cardiovascular Medicine University of Utah Health Salt Lake City Utah USA; ^2^ Department of Internal Medicine University of Utah Health Salt Lake City Utah USA

**Keywords:** anti‐arrhythmic drugs, atrial fibrillation, hemodynamic effects, lidocaine, pro‐arrhythmia, ventricular arrhythmia

## Abstract

**Introduction:**

Intravenous lidocaine is frequently used for refractory ventricular arrhythmia (VA). Atrial fibrillation (AF) is a common comorbidity in VA patients. Lidocaine poses theoretical risks of hemodynamic compromise and pro‐arrhythmia, including AF with rapid ventricular rates (RVR).

**Methods:**

This observational study reviewed electronic medical records of AF patients receiving lidocaine for VA at University of Utah Hospital (April 2014–October 2023). Hemodynamic parameters and cardiovascular outcomes were analyzed.

**Results:**

192 patients (mean age 63.6 years, 79% male) with predominantly paroxysmal AF (71%) were included. 79% had heart failure (HF), 53% ischemic, with a mean ejection fraction 31.7%. There was no difference in heart rate (−0.6 ± 31.6; *p* = 0.4067), but systolic blood pressure and mean arterial pressure decreased on lidocaine vs baseline (−4.7 ± 22.6; *p* = 0.0367 and −4.8 ± 18.6; *p* = 0.0013, respectively). A single patient had AF with RVR attributed to lidocaine with no significant difference in AF with RVR on baseline vs lidocaine ECG (16, 9% vs 8, 6%; *p* = 0.3957). Peak lidocaine levels (median 4.3 mcg/mL; IQR 3.0–5.8 mcg/mL) were not correlated with RVR (*p* = 0.9662). Adverse effects occurred in 7%, mainly central nervous system effects, with no clinically significant hypotension or worsening HF. In‐hospital mortality was 31%. Ischemic cardiomyopathy vs non‐ischemic cardiomyopathy was associated with increased mortality (*p* = 0.0162).

**Conclusion:**

Lidocaine for VA in AF patients had mild hemodynamic impacts without clinically significant hypotension or worsening HF. The risk of AF pro‐arrhythmia was low. Lidocaine use was associated with a high‐risk population with high in‐hospital mortality.

**Abbreviations**
AF: atrial fibrillation
CNS: central nervous system
ECG: electrocardiographic
HF: heart failure
HR: heart rate
IV: intravenous
MAP: mean arterial pressure
RVR: rapid ventricular rate
SBP: systolic blood pressure
VA: ventricular arrhythmia

## 1 Introduction

Lidocaine is a class Ib anti‐arrhythmic drug that acts on sodium channels to reduce the action potential duration and increase the refractory period, making it useful in the management of reentrant rhythms [1, 2, 3, 4, 5, 6]. It was initially developed as a local anesthetic and intravenous (IV) lidocaine was first administered as an anti‐arrhythmic drug in 1950 [2, 7]. For a time, it was a first‐line agent for ventricular arrhythmias (VAs), however safety concerns have resulted in more limited use over time [1]. A recent large retrospective study of patients receiving either amiodarone or lidocaine for ventricular tachycardia or ventricular fibrillation in‐hospital cardiac arrest revealed significantly greater survival and favorable neurologic outcomes with lidocaine compared to amiodarone [8]. Current guidelines recommend lidocaine for the management of cardiac arrest due to refractory ventricular fibrillation or polymorphic ventricular tachycardia [9].

When given rapidly, lidocaine can result in dose‐dependent reductions in ventricular contractility, arterial pressure, and heart rate (HR) [1, 2, 10, 11, 12]. Atrial fibrillation (AF) and heart failure (HF) have a bidirectional association and both can contribute to susceptibility to hemodynamic and electrical instability. Acceleration of ventricular rates in AF has been reported with lidocaine administration [2, 13]. Concern for development of AF with rapid ventricular rates (RVR) and hemodynamic instability has resulted in caution to use lidocaine in this patient population.

Increasingly, lidocaine infusion has alternative uses, including the management of acute and chronic pain. Recent studies have shown promising results in the treatment of neuropathic pain, chronic post‐surgical pain, fibromyalgia, cancer pain, and complex regional pain syndrome [14]. Often, patients in these groups are older and as such experience higher rates of AF. Limited clinical experience and concerns about adverse effects in patients with AF, alongside the largely elective nature of its newer alternative indications, often restrict lidocaine's use in this population

Despite relatively common use of lidocaine in patients with refractory VA and cardiac comorbidities such as AF and HF in real world clinical practice, there is little data describing outcomes of lidocaine administration in the contemporary era. We sought to evaluate the hemodynamic effects and clinical outcomes in patients with AF receiving lidocaine infusion for VA suppression.

## 2 Materials and Methods

This was a retrospective, observational study of patients with AF who received IV lidocaine for VA management at the University of Utah Hospital from April 2014 to October 2023. The study included adults aged 18 years or older with a confirmed 12‐lead‐electrocardiographic (ECG) diagnosis of AF who were administered IV lidocaine with a bolus and/or continuous infusion for VA during the study period.

Baseline demographics, medical comorbidities, medication administration records, vital signs, hemodynamic data, laboratory parameters, ECG data, and echocardiographic data were reviewed and collected. Outcomes including lidocaine adverse effects, coronary revascularization, VA ablation, stellate ganglion block, sympathectomy, cardiac transplantation, all cause death, and death from cardiovascular causes were recorded and analyzed. Clinical lidocaine adverse events were categorized as central nervous system (CNS) effects, bradycardia, arrhythmia (including AF with RVR), hypotension, and worsening HF. AF with RVR was defined as AF with a heart rate (HR) greater than 100 bpm on a 10 s ECG or sustained AF with a HR greater than 100 bpm observed on telemetry monitoring and reported in provider documentation during the admission. Baseline hemodynamic parameters, including HR, systolic blood pressure (SBP), and mean arterial pressure (MAP) while receiving lidocaine were compared with baseline hemodynamic data prior to lidocaine initiation. The associations between medical comorbidities, hemodynamic parameters, ECG data, and patient outcomes were analyzed using *X* ^2^ and Fisher's exact test for categorical variables. The *t*‐test was used for comparison of continuous variables. Normality was tested, and non‐normally distributed continuous variables were evaluated with the Wilcoxon signed‐rank test. Data analysis was performed using JMP statistical analysis software Version 18 (SAS Institute, Cary, NC).

This retrospective research protocol was approved by the institutional review board of University of Utah Health, which waived the need for informed consent as the research presented minimal risk to the participants and the waiver did not adversely affect their rights or welfare.

## 3 Results

### 3.1 Patient Characteristics

There were 192 patients with a history of AF who received lidocaine for VA (mean age 63.6% ± 12.2 years; 79% male; mean BMI 30.5 ± 6.9 kg/m^2^; 65% hypertension; 42% diabetes; 62% coronary artery disease; 79% HF; mean EF 31.7% ± 16.1%) (Table 1).

**Table 1:** Baseline demographics of study patients with atrial fibrillation who received lidocaine for ventricular arrhythmias.

	*N* = 192
Age (years)	63.6 ± 12.2
Male	152 (79%)
Race	
White	146 (76%)
Black	3 (2%)
Asian	1 ( < 1%)
Native American	7 (4%)
Hispanic	12 (6%)
Unknown	23 (12%)
BMI (kg/m^2^)	30.5 ± 6.9
AF classification	
Paroxysmal	135 (71%)
Persistent	48 (25%)
Permanent	8 (4%)
Hypertension	123 (65%)
Diabetes	79 (42%)
Coronary artery disease	118 (62%)
Heart failure	150 (79%)
Left ventricular EF (%)	31.7 ± 16.1
Renal disease	39 (20%)
Liver disease	19 (10%)
Beta blocker use	132 (69%)
Amiodarone use	
Intravenous	153 (83%)
Oral	103 (54%)

Abbreviations: BMI, body mass index; EF, ejection fraction.

Values are number (%), mean ± 1 standard deviation.

The majority had paroxysmal AF (135, 71%) followed by persistent (48, 25%). 132 (69%) were on beta blockers. 153 (83%) received concurrent IV amiodarone and 103 (54%) transitioned to oral amiodarone. All patients received lidocaine infusions and the majority (147, 77%) were initiated with bolus doses ranging from 32 mg to 250 mg. Lidocaine drip rates ranged from 0.3 mg/min to 4.0 mg/min with most patients receiving 1.0 mg/min. Most ultimately stopped lidocaine without transition to an alternate class I drug (144, 76%) and 45 (24%) were transitioned to mexiletine.

The number of lidocaine levels checked during the infusion ranged from 0 to 14 (median 2). In the majority of patients (151, 79%) a lidocaine level was checked approximately 24 h after initiation of the lidocaine infusion with repeat levels often drawn at 12–24 h intervals thereafter. The timing of lab draws was not standardized due to the retrospective nature of this analysis.

### 3.2 Electrocardiographic & Hemodynamic Changes

**Figure 1:**
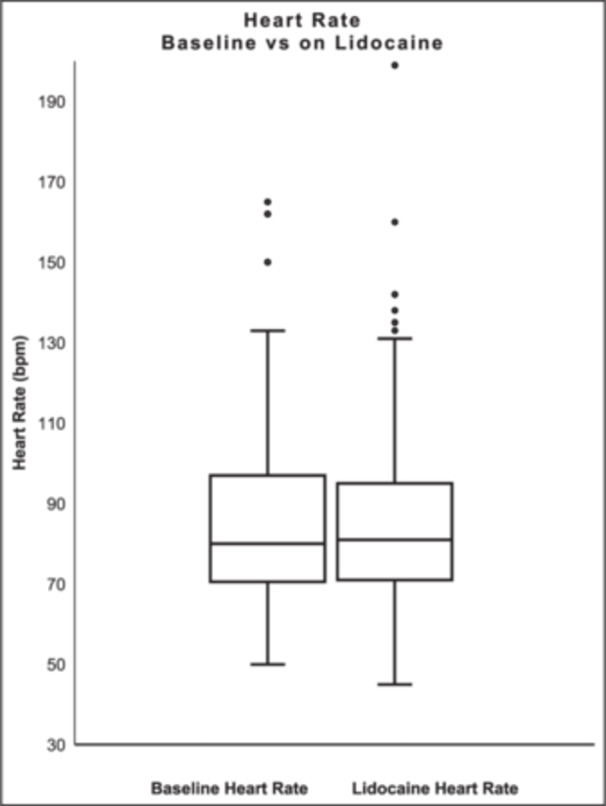
Box plot comparing heart rate at baseline and while on lidocaine.

**Figure 2:**
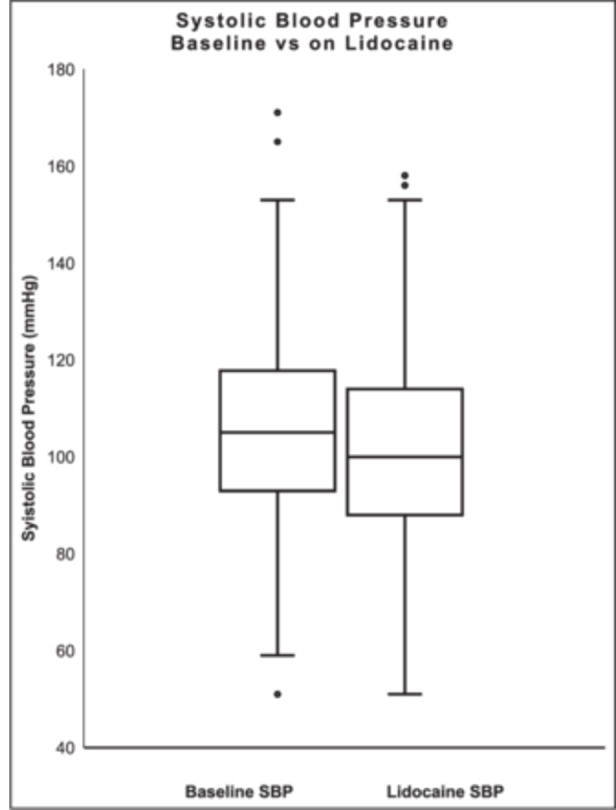
Box plot comparing systolic blood pressure (SBP) at baseline and while on lidocaine.

**Figure 3:**
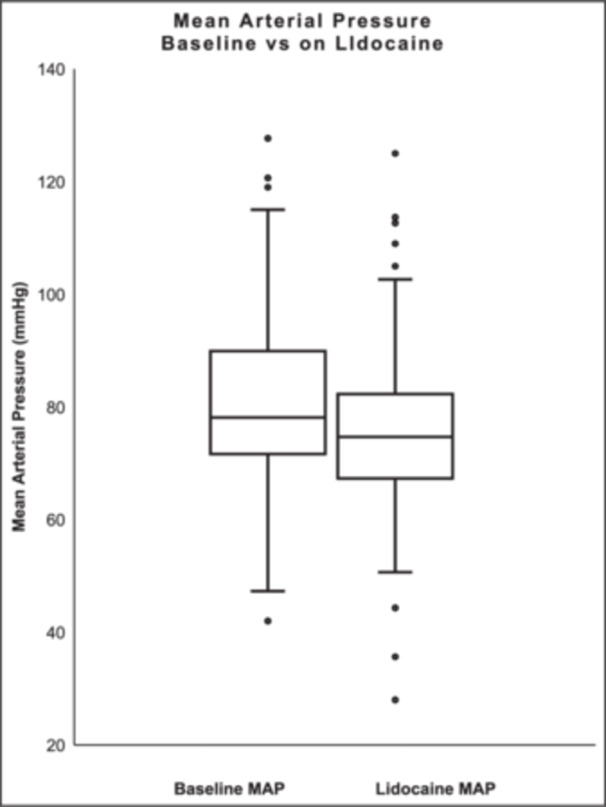
Box plot comparing mean arterial pressure (MAP) at baseline and while on lidocaine.

Admission mean HR was 86.6 ± 23.1 bpm, SBP 105.9 ± 19.9 mmHg, and MAP 80.7 ± 15.0 mmHg. On lidocaine the mean HR was 84.7 ± 21.7 bpm, SBP 101.6 ± 20.3 mmHg, and MAP 76.0 ± 13.3 mmHg. There was no significant change in the mean HR (−0.6 ± 31.6; *p* = 0.5751) (Figure 1). The mean SBP and MAP decreased after the administration of lidocaine (−4.7 ± 22.6; *p* = 0.0159 and −4.8 ± 18.6; *p* = 0.0011, respectively) (Figures 2, 3).

Prior to lidocaine initiation the rhythm was sinus in 81 (45%), ventricular paced in 53 (28%), AF in 31 (17%), and VT in 13 (7%). Of the 133 patients who had an ECG performed while receiving lidocaine, the rhythm was sinus in 63 (47%), ventricular paced in 36 (27%), AF in 19 (14%), and VT in 12 (9%).

Half of patients were reported to have experienced AF with RVR during admission based on chart review, though the majority of these episodes do not appear to have been contemporaneous with lidocaine administration. At baseline, 16 of the 31 (52%) patients in AF had RVR on ECG, accounting for 9% of the study population with ECGs available. After being placed on lidocaine 8 of the 19 (42%) patients in AF had RVR on ECG, for a total of 6% of those with ECGs available. There was no significant difference in the overall proportion of patients in AF with RVR on ECG prior to lidocaine administration compared with on lidocaine (*p* = 0.3957). Patients with AF who were on a beta‐blocker were less likely to develop RVR (53, 40% vs 34, 59%; *p* = 0.0157). This effect was predominantly seen in patients classified as having persistent AF (12, 31% vs 7, 78%; *p* = 0.0136) rather than paroxysmal (40, 47% vs 26, 54%; *p* = 0.2721).

At baseline, there were 53 (28%) who were ventricular paced, 14 (8%) who conducted with a right bundle branch block (RBBB), and 13 (7%) who conducted with a left bundle branch block (LBBB). On lidocaine, there were 36 (27%) who were ventricular paced, 11 (8%) who conducted with a RBBB, and 11 (8%) who conducted with a LBBB. There was no significant difference in the proportion of patients who were ventricular paced or who had aberrant ventricular conduction at baseline compared with on lidocaine (*p* = 0.7043 and *p* = 0.7541). There was no significant change in the average QRS duration (134.5 ± 42.2 vs 141.3 ± 58.9 ms) or QTc (493.2 ± 64.9 vs 506.6 ± 70.9 ms) on lidocaine compared with baseline (*p* = 0.5722 and *p* = 0.2376, respectively). There remained a non‐statistically significant increase in QRS duration observed when individuals with ventricular pacing and ventricular tachycardia were excluded (Table 2).

**Table 2:** Electrocardiographic intervals at baseline compared with on lidocaine.

	Baseline QRS duration (ms)	QRS duration on lidocaine (ms)	*p*‐value
All patients	134.5 ± 42.2	141.3 ± 58.9	0.5722
All except ventricular paced	123.4 ± 35.5	134.2 ± 58.0	0.1953
All except ventricular paced and ventricular tachycardia	116.2 ± 28.3	126.8 ± 53.5	0.6443
Ventricular paced only	172.2 ± 35.2	162.4 ± 33.3	0.2248

Values are number (%), mean ± 1 standard deviation.

**Figure 4:**
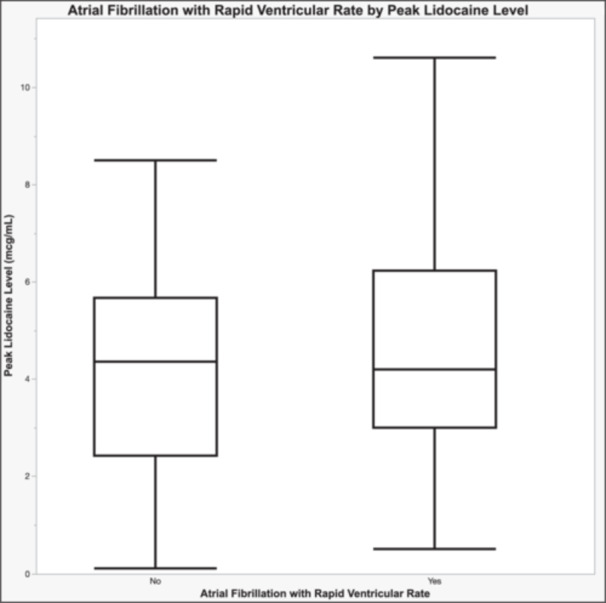
Box Plot comparing risk of rapid ventricular rates during atrial fibrillation with peak lidocaine levels.

Overall lidocaine levels ranged from 0.0 to 48.0 mcg/mL with a median of 3.3 mcg/mL (IQR 2.4–4.6 mcg/mL). Peak lidocaine levels ranged from 0.1 to 48.0 mcg/mL with a median of 4.3 mcg/mL (IQR 3.0–5.8 mcg/mL) and there was no correlation of risk of RVR with peak lidocaine level (*p* = 0.9662; Figure 4).

### 3.3 Lidocaine Adverse Effects

Adverse effects attributed directly to lidocaine occurred in 13 patients (7%) and were predominantly CNS effects (10, 5%). There were 2 patients (1%) who developed bradycardia during infusion. In one patient, bradycardia occurred in the context of concurrent use of a beta blocker and amiodarone, with all potentially offending medications subsequently held. The second patient was administered simultaneous lidocaine and amiodarone, and both were held due to a junctional rhythm. A single individual (< 1%) experienced AF with RVR attributed to IV lidocaine based upon medical record documentation.

### 3.4 Cardiovascular Outcomes

**Figure 5:**
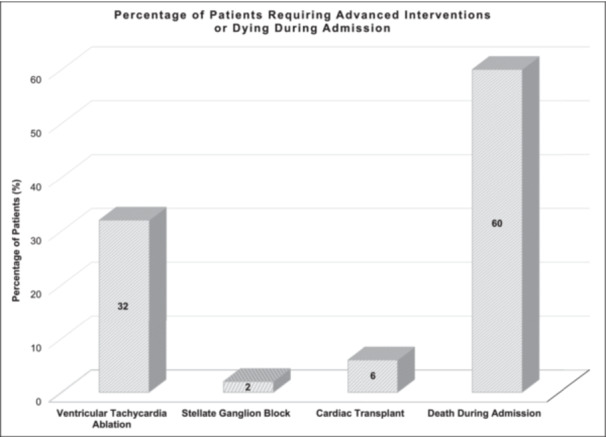
Bar graph showing percentage of patients with atrial fibrillation receiving intravenous lidocaine who require advanced interventions or died during admission.

There were 32 (17%) patients who underwent VA ablation, 2 (1%) stellate ganglion block, 6 (3%) cardiac transplant, and 60 (31%) who died during hospital stay (Table 3, Figure 5). The cause of death was cardiogenic shock or VA in 31 (52%), acute coronary syndrome in 6 (10%), and non‐cardiac in 23 (38%). Male patients more often had VA ablation compared to female patients (31, 21% vs 1, 3%; *p* = 0.0029). There was a non‐significant trend toward lower mortality for VA ablation (6, 19% vs 53, 33%; *p* = 0.0906).

**Table 3:** Outcomes during the index hospitalization in patients with atrial fibrillation who received lidocaine for ventricular arrhythmia.

	*N* = 192
Coronary revascularization	57 (30%)
Ventricular arrhythmia ablation	32 (17%)
Stellate ganglion block	2 (1%)
Sympathectomy	0 (0%)
Cardiac transplantation	6 (3%)
In‐hospital death from any cause	60 (31%)
In‐hospital death from cardiovascular cause	37 (19%)

After VA ablation, patients were more often transitioned to mexiletine (12, 27% vs 18, 13%; *p* = 0.0243). Those in whom lidocaine was stopped, rather than transitioned to mexiletine, died more frequently (53, 37% vs 5, 11%; *p* = 0.0006). Of the 144 patients in whom lidocaine was stopped, 75 (52%) died from any cause, and 31 (22%) died related to a ventricular arrhythmia. There were 45 patients who were transitioned to mexiletine, and of those 17 (38%) died from any cause and 4 (9%) died related to a ventricular arrhythmia.

Patients with ischemic cardiomyopathy were more likely to die during admission than non‐ischemic (28, 35% vs 10, 17%; *p* = 0.0162). There were 14 (50%) patients with ischemic cardiomyopathy whose cause of death was cardiogenic shock or VA, 3 (11%) acute coronary syndrome, and 11 (39%) from non‐cardiac causes. In non‐ischemic cardiomyopathy the cause of death was cardiogenic shock or VA in 7 (70%) and non‐cardiac in 3 (30%).

### 3.5 Heart Failure Subgroup

A subset of 150 (72%) patients carried a clinical diagnosis of HF (mean age 63.4±12.2 years; 81% male; mean BMI 30.3 ± 6.2 kg/m^2^; 62% hypertension; 42% diabetes; 63% coronary artery disease). Of those with HF, 9 (6%) had preserved EF, 80 (53%) had ischemic cardiomyopathies, 58 (39%) non‐ischemic cardiomyopathies, and 2 (1%) mixed cardiomyopathies with a HF group mean EF of 28.0±13.5% (Table 4).

**Table 4:** Baseline demographics of the heart failure patient subgroup.

	*N* = 150
Age (years)	63.4 ± 12.2
Male	122 (81%)
Race	
White	117 (78%)
Black	2 (1%)
Asian	1 (0.7%)
Native American	5 (3%)
Hispanic	8 (5%)
Unknown	17 (11%)
BMI (kg/m^2^)	30.3 ± 6.2
AF classification	
Paroxysmal	96 (64%)
Persistent	47 (31%)
Permanent	7 (5%)
Hypertension	93 (62%)
Diabetes	62 (42%)
Coronary artery disease	94 (63%)
Heart failure etiology	
Preserved EF	10 (7%)
Ischemic	79 (53%)
Non‐ischemic	59 (39%)
Mixed	2 (1%)
Left ventricular EF (%)	28.0±13.5
Beta blocker use	110 (74%)
Amiodarone use	
Intravenous	116 (77%)
Oral	93 (62%)

Abbreviations: BMI, body mass index; EF, ejection fraction.

Values are number (%), mean ± 1 standard deviation.

At admission, the mean HR was 85.8 ± 22.6 bpm, SBP 105.5 ± 18.8 mmHg, and MAP 80.7 ± 14.2 mmHg. On lidocaine, the mean HR was 81.0 ± 17.9 bpm, SBP 99.1 ± 19.1 mmHg, and MAP 75.0 ± 12.9 mmHg. The HR, SBP, and MAP decreased on lidocaine (*p* = 0.0423, *p* = 0.0042, and *p* = 0.0003, respectively).

Clinically significant adverse effects related to lidocaine were identified in 10 (7%) patients, with central nervous system effects in 9 (6%) and pro‐arrhythmia in 1 (< 1%). None had hypotension or worsening HF attributed to lidocaine. There was no association between the occurrence of reported lidocaine adverse effects and EF (*p* = 0.4585).

There were 6 (4%) patients who underwent cardiac transplant and 41 (27%) who died. Those with ischemic cardiomyopathy were more likely to die during admission (28, 35% vs 10, 17%; *p* = 0.0162).

## 4 Discussion

Lidocaine is frequently used for the treatment of refractory VA, yet there is little modern data describing its use, safety, and outcomes. Concerns about potential adverse effects may limit its use for alternative indications in patients with AF.

Our study evaluated a cohort of patients with AF who received IV lidocaine for treatment of VA. The drug‐related adverse event rate was low with less than 1% of patients experiencing rapid AF attributable to lidocaine. We found lidocaine use to have a mild but statistically significant effect on hemodynamic parameters, including SBP and MAP, without any clinically significant hypotension. Despite its apparent safety, the need for lidocaine was associated with a high‐risk patient population with a high mortality rate.

The effect of lidocaine on atrial tissue is less than on Purkinje fibers and ventricular tissue [3, 4, 15, 16]. For this reason, it is not commonly used to treat atrial arrhythmias, though it may be given to patients with atrial arrhythmias during treatment of other disorders. One randomized study by Marrouche et al. investigated high‐dose bolus lidocaine for pharmacologic cardioversion of AF and demonstrated no significant effect for conversion to sinus rhythm [17].

AF with RVR has been reported with lidocaine administration [2, 13]. A 1978 study by Danahy and Aronow evaluated the effect of a 100 mg lidocaine dose on atrial flutter and fibrillation in 51 patients. They observed reduced atrial flutter rates with both slow and rapid bolus lidocaine administration with variable changes in the ventricular response. In AF, an increase in the mean ventricular rate by 6 bpm was observed [18].

We found no significant increase in the HR in all patients when comparing the baseline HR and the HR on lidocaine. There was a single case of AF with RVR attributed to lidocaine infusion, with an overall event rate of < 1% in this cohort. Our results suggest that lidocaine infusion is safe with a low risk of RVR in patients with AF even when used in the setting of structural heart disease.

Limitations to our study include the small sample size, predominantly white male population, and retrospective study design which limit the broader applicability and prevent causal associations. Additionally, it is noted that many patients were receiving other medical therapy such as beta blockers and amiodarone, which could reduce the risk of pro‐arrhythmia and impact vital signs. However, these medications are frequently necessary when lidocaine, a second‐line treatment for VAs, is administered. Lidocaine administration and testing were not standardized due to the retrospective nature of the study. Lack of standardization may impact the incidence of adverse events and potential efficacy of lidocaine therapy. Despite these limitations, these data are still compelling, particularly in consideration of drug administration in a complex patient population with multiple medical comorbidities, and suggest in aggregate a low risk of adverse events supporting the safety of lidocaine use in patients with AF.

## 5 Conclusion

In this cohort of patients with AF who received IV lidocaine for the management of VA, there was a mild hemodynamic impact observed during infusion without clinically significant instances of hypotension or worsening HF reported as a result. There was an overall low adverse event rate, including a less than 1% risk of AF pro‐arrhythmia. We found no association between the risk of AF with RVR and the peak lidocaine level. While lidocaine‐associated adverse events were uncommon, lidocaine use in this setting was associated with a high‐risk population with a third of patients dying during hospitalization.

## Funding

The authors received no specific funding for this work.

## Conflicts of Interest

ANK (none), ZA (none), DF (none), LS (none), MCE (none), RAF (none), KK (none), HIL (none), RR (none), BAS reports salary support from the NIH/NHLBI (#R56HL168264, #R21HL172288, #1R01HL177105), and research support from Abbott, Boston Scientific, Biosense‐Webster, Sanofi, and AltaThera; and consulting to Sanofi, Bayer, Boston Scientific, Element Science, Milestone, and AltaThera., TJB (none).
